# Weight-Independent Cardioprotective Effects of Physical Activity in Patients with Myocardial Infarction

**DOI:** 10.3390/diagnostics16203262

**Published:** 2026-10-09

**Authors:** Corina Cinezan, Camelia Bianca Rus, Timea Claudia Ghitea, Teodora Bianca Sirca-Tirla, Adrian Tirla

**Affiliations:** 1 Department of Medical Disciplines, Faculty of Medicine and Pharmacy, University of Oradea, 410073 Oradea, Romania; 2 Clinical County Emergency Hospital Bihor, 410169 Oradea, Romania; 3 Doctoral School of Biological and Biomedical Sciences, University of Oradea, 410087 Oradea, Romania; 4 Pharmacy Department, Faculty of Medicine and Pharmacy, University of Oradea, 410073 Oradea, Romania

**Keywords:** myocardial infarction, coronary artery disease, physical activity, body mass index, mediation analysis, coronary angiography, secondary prevention

## Abstract

**Background:** Physical activity is a key modifiable determinant of cardiovascular risk, yet whether its protective association with coronary artery disease (CAD) severity in patients with myocardial infarction (MI) is mediated by body mass index (BMI) remains uncertain. **Methods:** This cross-sectional study included 294 adults admitted with MI and angiographically confirmed MI. Daily preadmission physical activity, as a score, BMI, cardiovascular risk factors and angiographic CAD extent (single-, double-, or triple-vessel disease) were analyzed. Multivariable ordinal logistic regression assessed independent associations of preadmission physical activity with CAD severity. Mediation analysis evaluated BMI as an intermediary using non-parametric bootstrap resampling with 2000 iterations and 95% confidence intervals (CIs). **Results:** Higher preadmission physical activity was independently associated with lower CAD severity after adjustment for age, sex, smoking, hypertension, diabetes, hyperlipidemia and BMI (β = −0.54, *p* < 0.001). BMI showed no independent association with CAD severity (β = 0.009, *p* = 0.76). Bootstrapped mediation analysis demonstrated no significant indirect effect through BMI (mean indirect effect 0.00044; 95% CI −0.00184 to 0.00360). **Conclusions:** In patients with MI, the inverse relationship between physical activity and CAD severity appears independent of BMI, suggesting weight-independent cardioprotective mechanisms. Promotion of habitual physical activity should remain a central component of secondary cardiovascular prevention regardless of body weight.

## 1. Introduction

CAD and its most severe clinical manifestation, myocardial infarction, remain leading causes of morbidity and mortality worldwide despite substantial advances in prevention, diagnosis and treatment [1,2,3]. Lifestyle-related risk factors, particularly physical inactivity and obesity play central roles in the initiation and progression of atherosclerosis, plaque instability and subsequent coronary events. Consequently, contemporary cardiovascular prevention and secondary-prevention guidelines emphasize regular physical activity and weight management as core therapeutic strategies for patients at risk of or recovering from MI [4,5,6].

Extensive epidemiological and mechanistic evidence demonstrates that habitual physical activity is associated with reduced incidence of myocardial infarction, lower rates of recurrent ischemic events, improved endothelial function, decreased systemic inflammation and enhanced survival [5,7,8,9]. Beyond risk-factor modification, emerging imaging and angiographic data suggest that physical activity may also influence the structural severity and distribution of coronary atherosclerosis, which are key determinants of MI occurrence and prognosis [10,11].

BMI is widely used as a surrogate marker of adiposity and cardiometabolic risk in patients with CAD and prior MI. However, the relationship between BMI and coronary disease severity or post-MI outcomes is complex and sometimes paradoxical, with several studies reporting weak associations between BMI and angiographic disease burden as well as the so-called “obesity paradox” in established cardiovascular disease populations. These observations raise the possibility that the cardiovascular benefits of physical activity in MI patients may occur partly independent of body weight [12,13,14,15].

Clarifying whether BMI mediates the relationship between physical activity and angiographic CAD severity in individuals presenting with MI has important clinical implications. If the protective effect of physical activity is largely weight-independent, secondary-prevention strategies should prioritize improving habitual physical activity and cardiorespiratory fitness even in the absence of meaningful weight reduction [5,16,17].

Therefore, the aim of the present study was to evaluate the association between daily preadmission physical activity and angiographic severity of coronary artery disease in patients with myocardial infarction and to determine whether this relationship is mediated by BMI.

## 2. Materials and Methods

### 2.1. Study Design and Population

This cross-sectional observational study included 294 consecutive adult patients admitted with acute myocardial infarction at Clinical County Emergency Hospital Bihor, Department of Cardiology, between November 2025 and August 2026. All patients underwent diagnostic coronary angiography, performed with an Azurion 3 System (Philips Medical systems Nederland B.V., Best, The Netherlands, 2020), as a treatment strategy, with the intention of coronary stent implantation, if needed. Only individuals with angiographically confirmed coronary artery disease and complete clinical and anthropometric data were included in the final analysis.

#### 2.1.1. Inclusion Criteria

Patients were eligible if they: were aged ≥18 years; had a confirmed diagnosis of first myocardial infarction according to contemporary clinical, electrocardiographic and biomarker criteria, including both non-ST and ST-elevation myocardial infarction; underwent coronary angiography during the index hospitalization; had complete demographic, clinical, physical activity and BMI data available.

#### 2.1.2. Exclusion Criteria

Patients were excluded if they had: missing or incomplete key clinical variables; left main disease during the index hospitalization; prior myocardial infarction or myocardial revascularization; an inability to perform physical activity, like arthrosis, anemia, severe angina, severe heart failure and severe valvular disease; pulmonary hypertension; congenital heart disease; chronic liver disease; advanced chronic respiratory or renal disease; neoplasm; lacked BMI measurement or did not undergo diagnostic coronary angiography. Patients with non-interpretable or unavailable angiographic reports were also excluded.

#### 2.1.3. Clinical and Anthropometric Variables

Baseline variables included age, sex, smoking status, hypertension, diabetes mellitus, hyperlipidemia, BMI and daily preadmission physical activity.

BMI was calculated as weight in kilograms divided by height in meters squared (kg/m^2^).

#### 2.1.4. Assessment of Physical Activity

Daily preadmission habitual physical activity was assessed within the first 24 h after hospital admission using a structured investigator-administered questionnaire referring to the four weeks preceding myocardial infarction. The questionnaire evaluated habitual physical activity across multiple domains, including daily walking, stair climbing, household activities, occupational physical activity, sedentary behavior and participation in recreational exercise or physically active leisure activities.

Participants were instructed to consider their overall level of habitual physical activity during the preceding four weeks when answering the questionnaire. The individual items were intended to guide participants in estimating their usual activity level and were not scored individually. After completing the interview, each participant assigned a single global habitual physical activity score (HPAS) on a 10-point ordinal scale, where 1 represented a predominantly sedentary lifestyle with minimal physical activity and 10 represented very high habitual physical activity, including heavy manual labor or daily vigorous exercise. Intermediate values reflected progressively increasing levels of habitual physical activity.

The HPAS was analyzed as an ordered continuous variable, with higher scores indicating greater habitual physical activity. The questionnaire is provided as Supplementary S1.

#### 2.1.5. Assessment of Coronary Artery Disease Severity

Severity of CAD was determined from coronary angiography performed during the index MI hospitalization and categorized according to the number of significantly (over 70%) stenosed major epicardial vessels (left anterior descending, right coronary and circumflex artery): single-vessel disease, double-vessel disease and triple-vessel disease.

CAD severity was analyzed as an ordered categorical outcome.

### 2.2. Statistical Analysis

Continuous variables were summarized as mean ± standard deviation and categorical variables as counts and percentages.

The association between preadmission physical activity and angiographic CAD severity in MI patients was evaluated using multivariable ordinal logistic regression, adjusted for age, sex, smoking, hypertension, diabetes mellitus, hyperlipidemia and BMI.

Regression coefficients (β), representing log-odds estimates from the ordinal logistic regression model, together with two-sided *p*-values, were reported. Negative β coefficients indicate a lower likelihood of belonging to a more severe category of coronary artery disease. Statistical significance was defined as *p* < 0.05.

### 2.3. Mediation Analysis

A causal mediation framework was applied to determine whether BMI mediated the relationship between physical activity and CAD severity.

- Path a represented the association between physical activity and BMI.
- Path b represented the association between BMI and CAD severity after adjustment for physical activity.
- The indirect effect was calculated as the product of paths a × b.

Statistical significance of mediation was assessed using non-parametric bootstrap resampling (2000 iterations) to generate 95% confidence intervals. Mediation was considered present if the confidence interval did not include zero.

No formal sample size calculation was performed because this was a cross-sectional observational study, and all consecutive eligible patients admitted during the study period were included in the analysis.

Missing data were handled using complete-case analysis. Patients with missing values for any of the study variables were excluded before statistical analysis; therefore, no data imputation was performed.

### 2.4. Software

All statistical analyses were performed using Python 3.3 -based statistical libraries in accordance with contemporary epidemiologic and biostatistical standards.

## 3. Results

The study included 294 patients with angiographically confirmed CAD. Disease severity was categorized as single-, double-, or triple-vessel involvement and analyzed as an ordered outcome. Baseline clinical variables included age, sex, smoking status, hypertension, diabetes mellitus, hyperlipidemia, body mass index, daily preadmission physical activity and severity of CAD as shown in Table 1.

In unadjusted analysis, higher levels of daily preadmission physical activity were significantly associated with lower CAD severity (β = −0.15, *p* < 0.001).

To account for potential confounding, an ordinal logistic regression model was constructed including age, sex, smoking, hypertension, diabetes, hyperlipidemia, BMI and physical activity.

After multivariable adjustment, physical activity remained a strong independent predictor of lower CAD severity (β = −0.54, *p* < 0.001), whereas BMI was not independently associated with CAD severity (β = 0.009, *p* = 0.76).

Several traditional cardiovascular risk factors were independently associated with more severe CAD, including older age (*p* < 0.001), male sex (*p* = 0.002), smoking (*p* = 0.018), hypertension (*p* = 0.003), diabetes mellitus (*p* < 0.001) and hyperlipidemia (*p* < 0.001). These findings are shown in Table 2, consistent with established epidemiological evidence and support the internal validity of the dataset.

Mediation analysis was performed to determine whether BMI mediated the relationship between physical activity and CAD severity. Physical activity was not significantly associated with BMI (*p* = 0.67) and BMI was not associated with CAD severity after adjustment for activity (*p* = 0.28). Bootstrapped mediation using 2000 resamples demonstrated a mean indirect effect of 0.00044 with a 95% confidence interval ranging from −0.00184 to 0.00360, indicating no statistically significant mediated effect. These findings are shown in Table 3.

**Figure 1:**
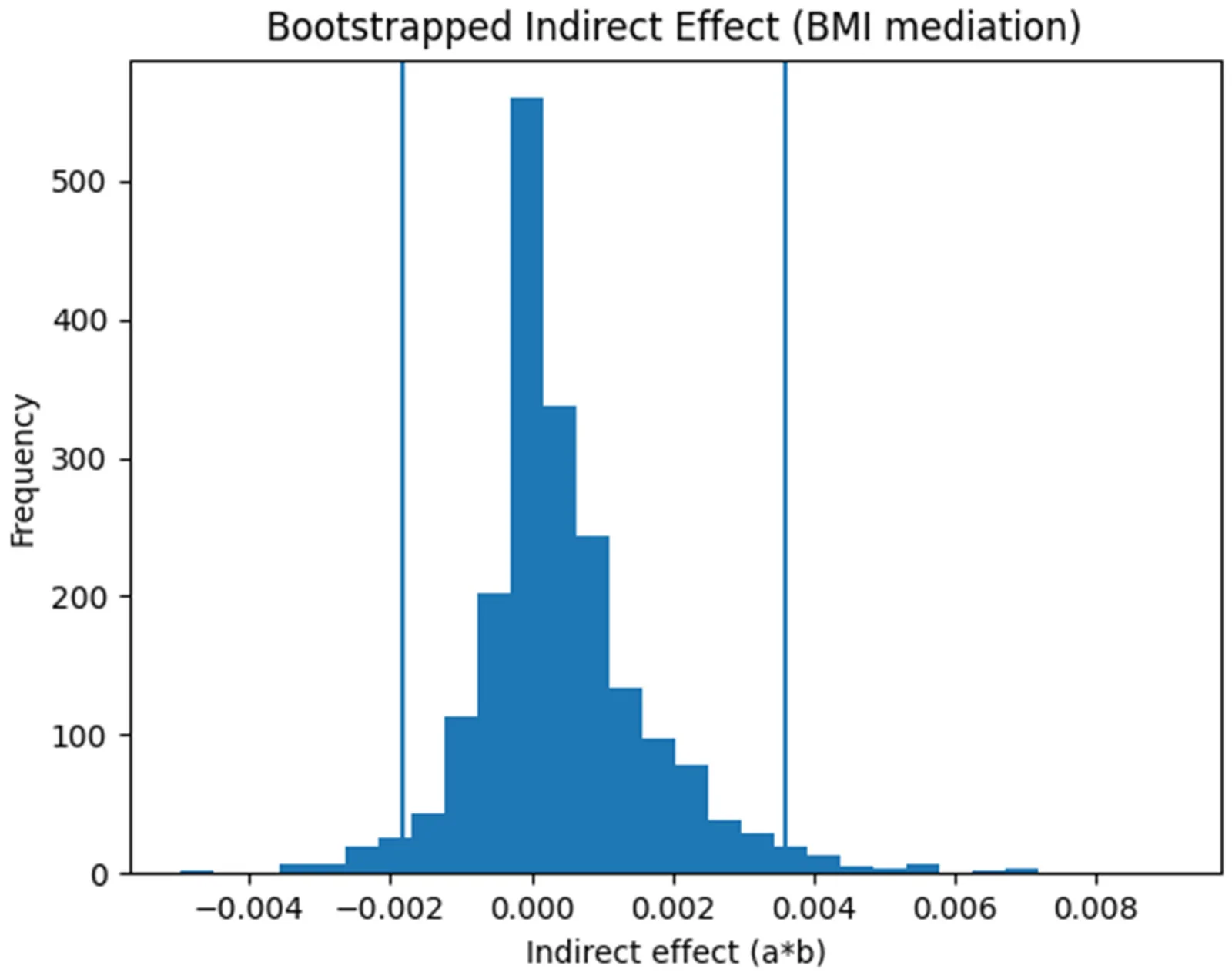
Bootstrapped indirect effect of BMI mediation.

Figure 1 presents the histogram illustrating the distribution of the bootstrapped indirect effect estimates (2000 resamples) for BMI as a mediator of the relationship between daily preadmission physical activity and angiographic coronary artery disease CAD severity in patients with myocardial infarction. The indirect effect was calculated as the product of path a (physical activity → BMI) and path b (BMI → CAD severity adjusted for physical activity and covariates). The vertical solid lines represent the 95% bootstrap confidence interval (−0.00184 to 0.00360). Because the confidence interval includes zero, no statistically significant mediation effect of BMI was observed.

**Figure 2:**
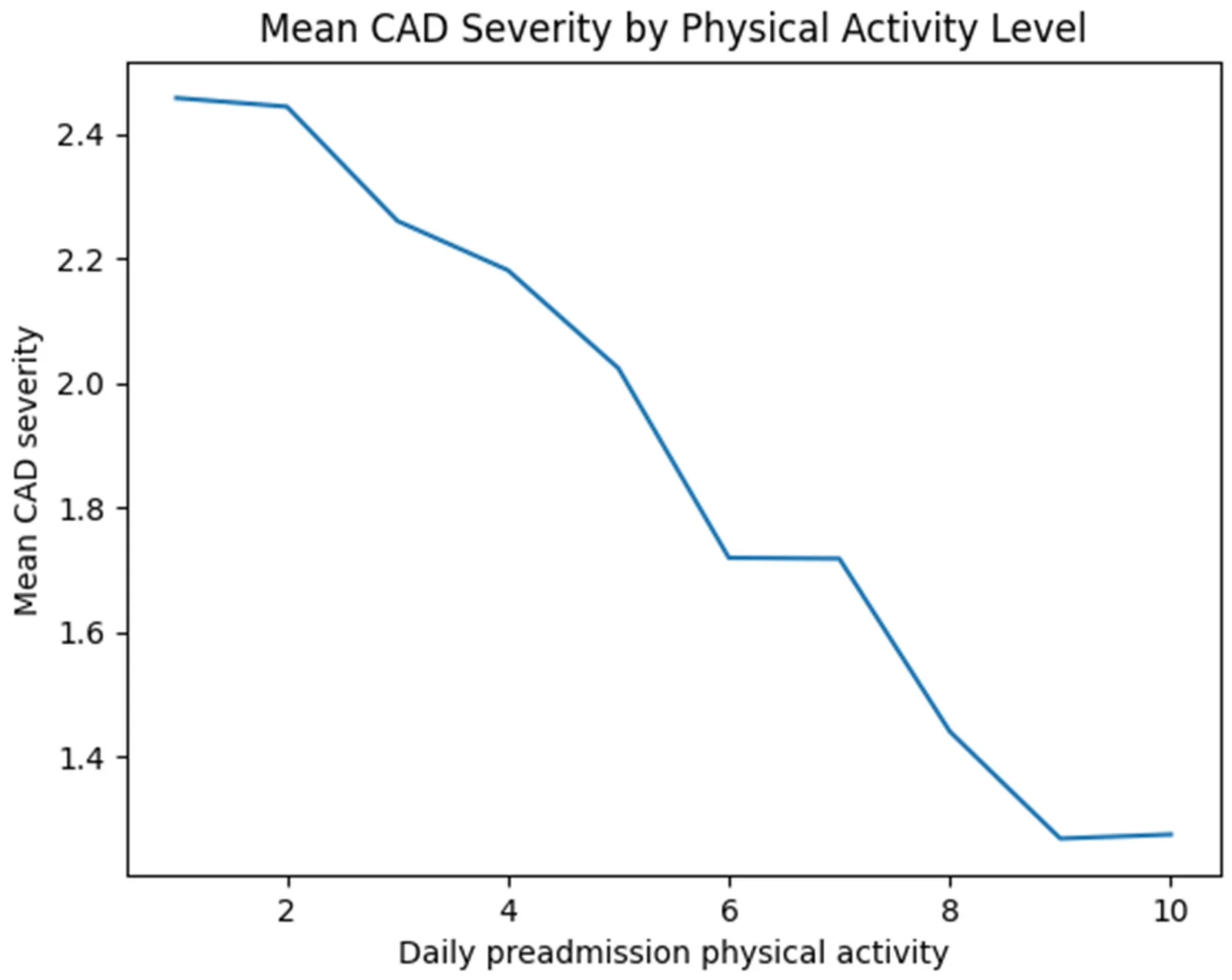
Mean CAD severity according to physical activity level.

Figure 2 illustrates the mean CAD severity across levels of daily preadmission physical activity in patients presenting with myocardial infarction. Physical activity was quantified using a global self-rated 1–10 score derived from the structured assessment of habitual activity during the preceding four weeks. Increasing activity levels were associated with progressively lower mean CAD severity (1 = single-vessel, 2 = double-vessel, 3 = triple-vessel disease). This figure is intended primarily as a descriptive visualization of the association observed in the present cohort rather than as evidence of a novel relationship between physical activity and coronary disease. The principal contribution of the present study is the evaluation of whether BMI mediates the association between physical activity and angiographic CAD severity in patients with myocardial infarction.

Overall, higher physical activity was strongly and independently associated with lower CAD severity, while BMI showed neither an independent association nor a mediating role. These findings suggest that the cardioprotective effect of physical activity operates through mechanisms other than BMI reduction.

## 4. Discussion

### 4.1. Principal Findings

In this cross-sectional study of patients presenting with myocardial infarction and angiographically confirmed coronary artery disease, higher levels of daily preadmission physical activity were strongly and independently associated with lower angiographic CAD severity. This association remained significant after adjustment for major cardiovascular risk factors, including age, sex, smoking, hypertension, diabetes mellitus, hyperlipidemia and BMI.

In contrast, BMI showed neither an independent relationship with CAD severity nor a mediating role in the association between physical activity and coronary disease burden. Bootstrapped mediation analysis confirmed the absence of a meaningful indirect pathway through BMI, indicating that the cardioprotective influence of habitual physical activity in MI patients operates largely through weight-independent mechanisms.

While the inverse association between physical activity and cardiovascular risk is well established, the present analysis specifically extends this evidence by examining BMI as a potential mediator of the relationship between habitual physical activity and angiographic CAD severity in patients presenting with myocardial infarction.

### 4.2. Comparison with the Previous Literature

The present study extends previous evidence linking habitual physical activity with cardiovascular protection by demonstrating an inverse and independent association between daily preadmission physical activity and angiographic CAD severity in patients presenting with MI. Importantly, this association persisted after adjustment for established cardiovascular risk factors, including age, sex, smoking, hypertension, diabetes mellitus, hyperlipidemia, and BMI. These findings are broadly consistent with the extensive epidemiological literature demonstrating that greater physical activity is associated with a lower risk of cardiovascular events and mortality, both in the general population and among individuals with established coronary artery disease [10,11,18,19]. An umbrella review incorporating systematic reviews and meta-analyses concluded that increasing moderate-to-vigorous physical activity is consistently associated with lower all-cause mortality, cardiovascular mortality, and incident atherosclerotic cardiovascular disease, including coronary heart disease [20]. Similarly, meta-analytic evidence has demonstrated a graded association between physical activity and cardiovascular outcomes, supporting the concept that even increases in activity among previously inactive individuals may provide clinically meaningful cardiovascular benefits [21].

Previous studies have suggested that physical activity may be associated not only with reduced cardiovascular event rates but also with more favorable coronary atherosclerotic characteristics. In a coronary computed tomography angiography study, [22] et al. reported an association between higher levels of exercise and differences in the extent, severity, and plaque characteristics of CAD. Similarly, observational and mechanistic studies have demonstrated that regular physical activity may influence several biological pathways involved in atherosclerosis, including endothelial function, vascular inflammation, oxidative stress, insulin sensitivity, and plaque stability [10,11]. More recently, an updated meta-analysis including 85 randomized controlled trials with coronary heart disease demonstrated that exercise-based cardiac rehabilitation was associated with a 26% reduction in cardiovascular mortality and an 18% reduction in myocardial infarction at the longest reported follow-up [23]. The present findings are compatible with these observations, as patients with higher habitual physical activity demonstrated lower angiographic CAD severity even after accounting for conventional cardiovascular risk factors. Nevertheless, because the present study is cross-sectional, the observed association should be interpreted as an independent relationship rather than evidence of a causal effect of physical activity on coronary atherosclerosis.

The strength of the present association is also noteworthy in comparison with studies examining individual cardiovascular risk factors. Traditional risk factors such as smoking, hypertension, diabetes, and dyslipidemia are well-established determinants of atherosclerotic disease and are incorporated into contemporary cardiovascular risk-prevention strategies [5,6]. Nevertheless, physical activity may represent a broader determinant of cardiovascular health that acts through multiple biological pathways simultaneously. Exercise can improve endothelial function, reduce systemic inflammation, improve glucose and lipid metabolism, enhance autonomic function, and increase cardiorespiratory fitness, potentially contributing to reduced progression or greater stability of atherosclerotic disease. The persistence of the association after adjustment for multiple conventional risk factors in the present study therefore supports the concept that the relationship between physical activity and coronary disease burden cannot be explained solely by differences in traditional risk-factor profiles [10].

An important finding of the present study was the absence of an independent association between BMI and angiographic CAD severity. This result is consistent with previous investigations reporting a relatively weak or inconsistent relationship between BMI and the anatomical extent of coronary atherosclerosis [12,24]. BMI is a convenient and widely available measure, but it does not distinguish between fat mass and lean mass, nor does it adequately capture visceral adiposity, body-fat distribution, or metabolic health [25]. Consequently, individuals with identical BMI values may have substantially different cardiometabolic profiles and levels of cardiovascular risk. In this context, bioelectrical impedance analysis (BIA) may provide complementary information beyond BMI by allowing estimation of body-composition parameters such as fat mass, fat-free mass, body cell mass, and total body water. BIA has been increasingly investigated in cardiovascular populations and may provide a more detailed characterization of body composition and nutritional status than BMI alone [26]. However, BIA also has methodological limitations, as its estimates may be influenced by hydration status, measurement conditions, and the prediction equations applied [27]. These considerations may be particularly relevant in patients hospitalized with acute myocardial infarction, in whom acute illness and changes in fluid balance may affect impedance-derived measurements. In the present study, BIA was not included in the predefined assessment protocol, whereas height and weight required for BMI calculation were routinely and consistently available for the entire study population. Future prospective studies incorporating standardized BIA measurements may help determine whether specific body-composition parameters provide additional information beyond BMI regarding the relationship between habitual physical activity and coronary artery disease severity.

Beyond total adiposity, the distribution of adipose tissue may be particularly relevant to coronary atherosclerosis. Visceral and ectopic adipose depots are metabolically active and may provide information that is not captured by BMI alone. In particular, epicardial adipose tissue has attracted increasing interest because of its anatomical proximity to the coronary arteries and its potential relationship with coronary atherosclerosis. A recent systematic review and meta-analysis demonstrated associations between increased epicardial adipose tissue and adverse cardiovascular outcomes, including myocardial infarction and cardiac death [28]. New prospective evidence demonstrated that epicardial fat thickness increased progressively with angiographic CAD severity and was also associated with a greater ischemic burden [29]. These observations further emphasize that individuals with similar BMI values may differ substantially in adipose tissue distribution and cardiovascular risk. Although visceral adiposity, epicardial fat, and detailed metabolic phenotyping were not assessed in the present study, their inclusion in future prospective studies could help clarify whether specific adipose tissue compartments or metabolic characteristics contribute to the relationship between physical activity and coronary disease severity.

The absence of an association between BMI and angiographic CAD severity in the present study may therefore reflect, at least in part, the limitations of BMI as a measure of biologically relevant adiposity in patients with established CAD. Reference [30] reported that lower BMI was associated with the highest risks of total mortality, cardiovascular mortality and myocardial infarction, whereas overweight patients had the lowest cardiovascular mortality. Furthermore [31] et al. reported in a meta-analysis of 36 studies involving patients undergoing coronary revascularization that low BMI was associated with the highest risks of total mortality, cardiovascular mortality, and MI, whereas overweight patients had the lowest cardiovascular mortality. Similarly, a network meta-analysis including 65 studies and more than 865,000 patients after PCI or CABG found a U-shaped association between BMI and mortality, with overweight and obese categories demonstrating lower mortality than the normal-weight category [32]. More recent evidence has produced similar observations specifically in acute coronary syndrome. A systematic review and meta-analysis involving 24 studies found that obesity was paradoxically associated with lower risks of several adverse outcomes following acute coronary syndrome, including reinfarction, overall mortality, and major adverse cardiovascular events, whereas underweight was associated with substantially higher risk [33]. A separate meta-analysis of STEMI patients undergoing primary PCI similarly reported lower short-term mortality among overweight and obese patients compared with patients with normal BMI [34]. More recent evidence further highlights the complexity of the relationship between BMI and outcomes after myocardial infarction. In a large population-based cohort of 10,651 patients with acute myocardial infarction, Schmitz et al. reported a U-shaped association between BMI and long-term all-cause mortality, with the lowest mortality observed in the BMI range of 25–30 kg/m^2^ [35]. Notably, the association also varied according to age, further illustrating that BMI alone may provide an incomplete characterization of cardiovascular risk after myocardial infarction.

These findings also contribute to the ongoing discussion surrounding the so-called “obesity paradox” in patients with established cardiovascular disease. Several observational studies have reported that overweight or mildly obese patients with established CAD or heart failure may have similar or even better short-term outcomes than patients with normal or low BMI. Although the mechanisms underlying this phenomenon remain debated, potential explanations include differences in body composition, disease severity, nutritional status, reverse causation, and the limitations of BMI as an indicator of adiposity. The present study does not directly investigate the obesity paradox because its primary outcome was angiographic CAD severity rather than long-term mortality. Nevertheless, the absence of an independent relationship between BMI and angiographic disease burden is compatible with the broader observation that BMI alone may have limited ability to characterize cardiovascular risk once established coronary disease is present [36,37].

Our findings therefore support and extend the concept that the cardiovascular benefits of physical activity should not be evaluated exclusively through its effects on body weight. In patients with MI, increased habitual physical activity may be associated with a lower burden of coronary atherosclerosis through multiple weight-independent mechanisms. This distinction has important clinical implications. A patient who remains overweight or obese despite becoming more physically active should not be considered to have failed to obtain cardiovascular benefit simply because substantial weight reduction has not occurred. Contemporary secondary-prevention strategies should consequently continue to emphasize regular physical activity and improvement in cardiorespiratory fitness alongside, rather than solely through, weight management [6].

### 4.3. Clinical Implications

From a clinical perspective, these findings reinforce the central importance of habitual physical activity as a cornerstone of secondary prevention after myocardial infarction, irrespective of body weight or the achievement of weight reduction. Patients who do not experience meaningful decreases in BMI may still derive substantial anti-atherosclerotic and prognostic benefit from regular physical activity. Accordingly, clinical care should prioritize the assessment and promotion of physical activity and cardiorespiratory fitness, alongside, but not contingent upon, weight management. This paradigm supports a shift toward emphasizing functional capacity and metabolic health over body mass alone in the long-term management of patients with established coronary artery disease [38,39,40].

This paradigm supports emphasizing functional capacity, physical activity, and metabolic health alongside body-weight management in the long-term care of patients with established coronary artery disease, consistent with contemporary cardiac rehabilitation recommendations that incorporate physical activity, exercise training, weight management, and body-composition assessment as components of comprehensive secondary prevention [9].

### 4.4. Strengths and Limitations

This study has several important strengths. First, the analysis was restricted to patients presenting with myocardial infarction and angiographically confirmed coronary artery disease, allowing an objective assessment of coronary disease severity at the time of a clinically relevant event. Second, the statistical approach incorporated comprehensive adjustment for major cardiovascular risk factors together with the use of ordinal logistic regression, which is appropriate for modeling graded angiographic disease burden. Third, the application of bootstrapped causal mediation analysis provided a robust evaluation of whether body mass index explains the relationship between habitual physical activity and coronary disease severity, thereby offering mechanistic insight beyond conventional association testing.

Several limitations should also be acknowledged. The cross-sectional design precludes causal inference and does not allow determination of temporal relationships between physical activity, body mass index and coronary disease progression. Physical activity was self-reported and based on a locally developed semi-quantitative scale rather than objective monitoring; therefore, recall bias and misclassification are possible. Furthermore, the assessment referred to habitual physical activity during the four weeks preceding myocardial infarction. Although this relatively short recall period may facilitate recall of recent activity, it may not fully represent long-term habitual physical activity. Importantly, some patients may have experienced angina, dyspnea, fatigue, or other manifestations of evolving ischemic heart disease during this period and consequently reduced their usual activity before the index event. Therefore, reverse causation cannot be excluded, whereby greater underlying coronary disease severity may have contributed to lower reported preadmission physical activity rather than lower physical activity necessarily preceding the development of more extensive coronary disease. Future prospective studies using longer-term activity assessment and objective measures such as accelerometry would help address this limitation. Another limitation is that coronary artery disease severity was classified according to the number of significantly stenosed epicardial vessels rather than by using more comprehensive angiographic scoring systems such as the SYNTAX or Gensini scores. Although this simplified classification is clinically relevant and easily applicable, it may not fully capture the complexity and anatomical extent of coronary atherosclerosis.

In addition, body mass index does not capture body composition, including fat mass, fat-free mass, skeletal muscle mass, or adipose tissue distribution, factors that may be more closely related to atherosclerotic burden. Although bioelectrical impedance analysis could provide complementary information regarding body composition, standardized BIA measurements were not included in the predefined assessment protocol of the present study. Future prospective studies incorporating BIA or other validated measures of body composition may further clarify whether specific components of body composition contribute to the observed association between physical activity and CAD severity.

Finally, the single-center nature of the study may limit generalizability to broader or more diverse myocardial infarction populations.

## 5. Conclusions

Among patients with myocardial infarction, higher habitual physical activity is strongly associated with less extensive coronary artery disease, independent of BMI and traditional cardiovascular risk factors. The protective effect of physical activity appears to operate through weight-independent biological pathways, underscoring the central importance of promoting physical activity as a cornerstone of secondary cardiovascular prevention after MI.

## Figures and Tables

**Table 1:** Baseline Characteristics of the Study Population.

Characteristic	Value
Number of patients	294
Age, mean ± SD (years)	59.1 ± 14.4
Male sex, *n* (%)	133 (45.2%)
BMI, mean ± SD (kg/m^2^)	27.0 ± 4.1
Smoking, *n* (%)	132 (44.9%)
Hypertension, *n* (%)	106 (36.1%)
Diabetes mellitus, *n* (%)	82 (27.9%)
Hyperlipidemia, *n* (%)	109 (37.1%)
Daily preadmission physical activity, mean ± SD (1–10)	5.63 ± 2.76
Single-vessel disease, *n* (%)	116 (39.5%)
Double-vessel disease, *n* (%)	101 (34.4%)
Triple-vessel disease, *n* (%)	77 (26.2%)

Notes: Continuous variables are presented as mean ± standard deviation; categorical variables as number (percentage).

**Table 2:** Multivariable Ordinal Logistic Regression Predicting CAD Severity.

Variable	β Coefficient	*p*-Value
Daily preadmission physical activity	−0.54	<0.001
BMI	0.009	0.76
Age	0.054	<0.001
Male sex	0.776	0.002
Smoking	0.603	0.018
Hypertension	0.784	0.003
Diabetes	1.228	<0.001
Hyperlipidemia	0.973	<0.001

Notes: Negative β values indicate association with lower CAD severity. Model adjusted for all listed covariates.

**Table 3:** Mediation Analysis of BMI in the Association Between Physical Activity and CAD Severity.

Parameter	Estimate
Indirect effect (mean)	0.00044
95% CI lower bound	−0.00184
95% CI upper bound	0.00360

Notes: Indirect effect estimated using bootstrap resampling (2000 samples). Confidence interval including zero indicates no significant mediation.

## Data Availability

The raw data supporting the conclusions of this article will be made available by the authors upon request. The original contributions presented in this study are included in the article. Further inquiries can be directed to the corresponding author.

## Supplementary Materials

The following supporting information can be downloaded at: https://www.mdpi.com/article/10.3390/diagnostics16203262/s1, S1: Structured Questionnaire for Assessment of Habitual Physical Activity During the Four Weeks Preceding Myocardial Infarction.
