# Supplementary material for: Weight-Independent Cardioprotective Effects of Physical Activity in Patients with Myocardial Infarction
Source: Diagnostics (Basel). 2026 Oct 9;16(20):3262. doi: 10.3390/diagnostics16203262 (PMC13703440; doi:10.3390/diagnostics16203262)
Supplement: Supplementary file 1 [file diagnostics-16-03262-s001.zip › diagnostics-4585183-supplementary.pdf]

Supplementary S1

Structured Questionnaire for Assessment of Habitual Physical Activity During the Four Weeks Preceding Myocardial Infarction

Patient ID: ______________________

Date: ____ / ____ / ______

Instructions

Please answer the following questions according to your usual level of physical activity during the four weeks before your current hospitalization.

Consider all types of physical activity, including your occupation, walking, stair climbing, household activities, transportation, gardening, recreational activities, and exercise.

There are no right or wrong answers. Please select the response that best reflects your usual activity.

Section 1. Daily Physical Activity

1. How often did you walk continuously for at least 15 minutes?

☐ Never

☐ Rarely (1–2 times/week)

☐ Occasionally (3–4 times/week)

☐ Frequently (5–6 times/week)

☐ Daily

2. How often did you choose stairs instead of an elevator whenever possible?

☐ Never

☐ Rarely

☐ Occasionally

☐ Frequently

☐ Almost always

3. How often did you perform household activities such as cleaning, cooking, shopping, gardening, or carrying groceries?

☐ Never

☐ Rarely

☐ Occasionally

☐ Frequently

☐ Daily

Section 2. Occupational Physical Activity

4. Which best describes your usual work or daily routine?

☐ Mostly sitting

☐ Mostly standing

☐ Walking for a substantial part of the day

☐ Moderate physical work

☐ Heavy manual labor

(If retired or unemployed, consider your usual daily activities.)

5. During a typical day, approximately how many hours did you spend sitting?

☐ More than 8 hours

☐ 6–8 hours

☐ 4–6 hours

☐ 2–4 hours

☐ Less than 2 hours

Section 3. Recreational Physical Activity

6. How often did you perform planned physical exercise (walking for exercise, cycling, swimming, running, gym training, or sports)?

☐ Never

☐ Less than once per week

☐ 1–2 days/week

☐ 3–4 days/week

☐ ≥5 days/week

7. How often did you participate in physically active leisure activities (gardening, hiking, dancing, active hobbies)?

☐ Never

☐ Rarely

☐ Occasionally

☐ Frequently

☐ Daily

Overall Physical Activity Rating

Considering all of your physical activities during the previous four weeks, please rate your overall habitual physical activity on the following scale.

Score	Description
**1**	Very low activity (predominantly sedentary lifestyle; almost no physical activity)
**2**	Very low activity with occasional walking or light household tasks
**3**	Low activity; light daily movement but mostly sedentary
**4**	Mildly active; regular walking but little moderate physical activity
**5**	Moderate activity; usual daily activities including walking and household tasks
**6**	Moderately active; frequent walking and regular moderate physical activity
**7**	Active; daily moderate physical activity or physically demanding occupation
**8**	Highly active; regular vigorous physical activity or heavy occupational work
**9**	Very highly active; vigorous physical activity on most days
**10**	Extremely active; heavy manual labor or daily vigorous exercise/sports

Global Habitual Physical Activity Score (HPAS)
Please circle one number that best represents your usual overall physical activity during the four weeks before hospitalization.

1 2 3 4 5 6 7 8 9 10

Investigator’s Note

The questionnaire items are intended to assist the patient in estimating their habitual physical activity during the four weeks preceding myocardial infarction. Individual responses are not scored separately. The single Global Habitual Physical Activity Score (HPAS; 1–10) assigned at the end of the interview is the variable used for statistical analyses, with higher scores indicating greater habitual physical activity.
